# A parasite through time: Revisiting *Trypanosoma rajae* Laveran and Mesnil, 1902 with new molecular and morphological insights from the blood of Rajidae in the western Mediterranean

**DOI:** 10.1016/j.ijppaw.2025.101097

**Published:** 2025-06-04

**Authors:** Sabrina Kefil, Linda Duval, Amandine Labat, Chahinez Bouguerche, Nadia Kechemir-Issad

**Affiliations:** a University of Algiers 1, Benyoucef Benkhedda, Faculty of Sciences, Department of Natural and Life Sciences, 2 Rue Didouche Mourad, 16000 Algiers, Algeria; b University of Science and Technology Houari Boumediene USTHB, Faculty of Biological Sciences, Biodiversity and Environment: Interactions-Genomes Laboratory, BP 32, El Alia Bab Ezzouar, Algiers, Algeria; c Unité de Communication Moléculaire et Adaptation des Microorganismes (MCAM, UMR7245), Muséum National d'Histoire Naturelle, CNRS, CP 52, 57 Rue Cuvier, 75005, Paris, France; d Department of Zoology, Swedish Museum of Natural History, Box 50007, SE-104 05, Stockholm, Sweden

**Keywords:** Kinetoplastida, Trypanosomatidae, *Trypanosoma rajae*, *Rajidae*, Mediterranean Sea, *18S rRNA*

## Abstract

Trypanosomes are blood parasitic protozoa infecting Chordates, including the elasmobranch skates (Rajidae). Amongst, *Trypanosoma rajae* Laveran and Mesnil, 1902 is a century old parasite, first briefly described from the Mediterranean starry ray *Raja asterias* and the undulate ray *R. undulata* off Roscoff, France, Northeast Atlantic, for which illustrations and molecular data are still lacking. Herein, we investigate blood trypanosomes of *R. asterias*, type-host of *T. rajae* and of those from the blood of the brown ray *R. miraletus*, collected off the Algerian coast, Western Mediterranean. We describe these trypanosomes using an integrative taxonomic approach combining morphological characters and partial 18S rRNA gene sequencing, and we provide several morphological, morphometrical, anatomical and biological characteristics (division stages). Specific characteristics of *Trypanosoma* ex *R. asterias* such as granulations of the nucleus and cytoplasm; shape of the kinetoplast, nucleus and body; tip of the body; the undulating membrane, as well as the morphometric measurements were within the ranges given in the previous records of *T. rajae*, and we ascribe thus the newly collected trypanosomes from *R. asterias* to *T. rajae*. Algeria and the Western Mediterranean are new locality records for *T. rajae*. Trypanosomes ex *R. miraletus* differed from *T. rajae* ex *R. asterias* by some morphometrical data: posterior end to kinetoplast (PK), free flagellum length (FF), the kinetoplast index (KI), slenderness (Sle), flagellar index (FI), mid-nucleus to anterior end (NA) and parasite maximum body width at nucleus (BWN). Partial 18S rRNA gene sequences of *T. rajae* ex *R. asterias* and *Trypanosoma* sp. ex *R. miraletus* showed ∼99.76 % similarity. We take a conservative position and refer to trypanosomes from *R. miraletus* as *T*. cf. *rajae*. Phylogenetic analysis using 18S rRNA gene sequences of other aquatic trypanosomes allowed positioning of *T. rajae* relative to the other trypanosome species, previously described, infecting marine and freshwater hosts worldwide. Some divisional stages were also observed on MGG-stained thin smears allowing a brief description of the division of this trypanosome in the blood of its host. This effort is the first study of *T. rajae* using integrative taxonomy combining morphology and DNA and we provide for the first time observations of dividing stages of this species in the host blood.

## Highlights

• This study provides the first integrative taxonomy of *Trypanosoma rajae.*
• *T. rajae* is newly reported from Algeria and the western Mediterranean.
• This study classifies trypanosomes from *Raja miraletus* as *T.* cf. *rajae.*
• We provide for the first time DNA barcodes, illustrations and data about *T. rajae* division stages in host blood.
• This study places *T. rajae* within the diversity of aquatic trypanosomes.

## Graphical abstract

**Figure:**
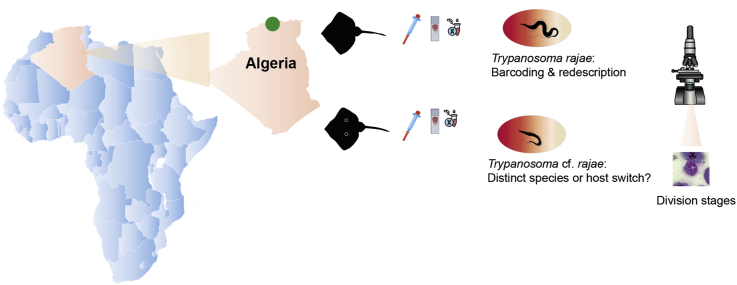

## 1 Introduction

The infection of marine and freshwater fish can be caused by blood parasites known as trypanosomes, hemoflagellates infecting vertebrate hosts, with many species capable of causing fatal infections in hosts, including fish and humans. The growth of high-density farming in both marine and freshwater aquaculture systems has led to an increase in severe outbreaks and fatalities due to trypanosomiasis. Investigating diversity of fish parasites and their associated pathologies are thus of importance to further manage fisheries. Among fish parasites, trypanosomes are transmitted to marine and freshwater fishes by leeches world-wide (Lom, 1979; Karlsbakk and Nylund, 2006). Compared to mammalian trypanosomes, the diversity of marine fish trypanosomes is yet to be investigated.

Early works dealing with rajid fish parasitic trypanosomes are those of Laveran and Mesnil (1902), Robertson (1907, 1909) and that of Kudo (1923) describing *Trypanosoma rajae* Laveran and Mesnil, 1901, *T. variabile* Neumann, 1909 and *T. scyllii* Laveran and Mesnil, 1902 from rajids collected in Atlantic waters, based on morphological data, hosts and locality. Based on the “one host-one species” paradigm, many *Trypanosoma* spp. were described with reference to their host i.e. *T. aeglefini* Henry, 1913 (note that currently this species is a *nomen nudum* (WoRMS, 2022)) from haddock *Melanogrammus aeglefinus* (Linnaeus) or *T. platessae* Lebailly, 1904 and *T. flesi* Lebailly, 1904, infecting plaice *Pleuronectes platessa* Linnaeus, and flounder *Platichthys flesus* (Linnaeus) (Burreson and Pratt, 1972; Burreson and Karlsbakk, 2007; Burreson, 2007). Although at least 100 trypanosome species infecting freshwater and marine fishes have been described, some of these species may be synonymous as they were established mainly based on host origin rather than parasite morphology, morphometrical or molecular data (Burreson and Pratt, 1972; Lom, 1979; Burreson, 1989; Lom and Dyková, 1992; Karlsbakk and Nylund, 2006; Burreson and Karlsbakk, 2007; Burreson, 2007; Gu et al., 2007). In recent years, several phylogenetic investigations have been conducted on freshwater fish trypanosomes (Corrêa et al., 2016; Smit et al., 2020; Aly and Ramadan, 2005; Hayes et al., 2006; Hayes et al., 2014; Zhang et al., 2023; Gu et al., 2006; Gu et al., 2007; Gibson et al., 2004; Smit et al., 2007) and marine fish trypanosomes (Lemos et al., 2015; Su et al., 2014; Karlsbakk and Nylund, 2006; Gu et al., 2010; Pretorius et al., 2021; Yeld and Smit, 2006; Hayes et al., 2014; Wang et al., 2016) contributing to a better understanding of this group of blood parasites.

One of the poorly known trypanosomes from marine fishes is the over a century old *T. rajae*, first described by Laveran and Mesnil (1902) from skates (Rajidae): the mediterranean starry ray *R. asterias* (syn. *R. punctata* Risso.), the undulate ray *R. undulata* (syn. *R. mosaica* Lacepède.) and additional hosts, mainly the thornback ray *R. clavata* Linnaeus and the common skate *Dipturus batis* Linnaeus (syn. *R. macrorynchus* Rafinesque), shortly after the original description (Laveran and Mesnil, 1904).

During an ongoing effort to explore the diversity of blood parasites of fishes of the Western Mediterranean, we investigated trypanosome occurrence in two species of Rajidae off the Algerian coast, *R. asterias* and the brown ray *R. miraletus* Linnaeus. Trypanosomes collected in the blood of *R. asterias* were consistent with the diagnosis of *T. rajae* and we ascribe them thus to the latter species. The lack of morphometrical and molecular data, and the brief original description of *T. rajae* prompts us to re-describe the species. We provide details about its biology and division stages observed in newly collected specimens. We also provide morphometrical and molecular data for *T.* cf. *rajae* from *R. asterias* and we discuss host specificity within skate blood trypanosomes.

## 2 Material and methods

### 2.1 Host collection

Between April 2011 to September 2012, and September 2013 to March 2016; 123 skates belonging to two species, *R*. *asterias* and *R. miraletus* (Rajiformes, Rajidae) were obtained from local fishermen in Bouharoune and Cap Djinet, Algeria, Western Mediterranean. Fishes were transferred to the laboratory shortly after capture, identified using morphological keys and examined on the day of purchase (Fischer et al., 1987; Pollerspöck and Straube, 2021). The length, width and sex of the fish hosts were determined and noted for all the examined specimens (Table 1).

**Table 1:** Infection rates, morphometrics, and sex distribution of the examined skates.

Hosts examined	Hosts infected	Average length	Average weight	sex	
*Raja asterias* (n = 73)	28	51.26 ± 1.64	961.26 ± 104.93	F:50	M:23
*Raja miraletus* (n = 50)	12	50.6 ± 1.63	798.72 ± 62.71	F:31	M:19

### 2.2 Morphological methods

Blood from fishes was obtained aseptically by heart puncture in the laboratory and examined fresh using light microscopy Nikon eclipse 50i. Thin smears (2–3 per fish) were prepared, air-dried, fixed with methanol, and stained with May-Grünwald Giemsa (MGG) and covered by coverslip with a few drops of Eukitt mounting medium. For each infected fish, air-dried smears were prepared without fixation or staining to allow for subsequent DNA extraction. Microscopic images of trypanosomes were captured by using a Nikon optical microscope with a Nikon digital camera DXm1200C. According to the terminology and morphometric values and standards commonly adopted by Karbowiak et al. (2005), 10 biometrical distances, proportions and indices were used to summarize the parasite morphology: body length (BL), posterior end to kinetoplast (PK), kinetoplast to mid-nucleus (KN), mid-nucleus to anterior end (NA), free flagellum length (FF), parasite maximum body width at nucleus (BWN), nucleus width (NuW), nucleus length (NuL), nucleus area (NuA), posterior end to mid-nucleus (PN), nuclear index (NI=PN/NA) (Dias and Freitas Filho, 1943). The kinetoplast index (KI=PN/KN)(Keymer, 1967), flagellar index (FI=FF/BL) and the slenderness (Sle = BL/BW) were also calculated (Borges et al., 2016). Measurements were produced with the “ImageJ software” (http://imagej.nih.gov/ij/), using a micrometric slide and the ImageJ software. Measurements are indicated as means (μm) ± standard deviation, the range and number of measurements. Drawings were made with the help of a Zeiss microscope with a drawing tube and digitized using a Wacom tablet and Adobe illustrator (2023).

### 2.3 Molecular methods

Total DNA was extracted from MGG-stained and unstained smears according to the manufacturer's instruction for Isolation of Genomic DNA from Swabs (QIAamp DNA Micro kit Handbook 08/2003). The presence of parasite DNA was assessed by PCR using specific primers of *Trypanosoma* 18S rDNA gene (Table 2) and followed by two overlapping nested-PCR (Maslov et al., 1996). Initial PCR and both nested-PCR were performed in 25 μL total volumes, including 2 μL of DNA or 2 μL of amplicon from the first reaction, 2 μL MgCl_2_ (25 mM), 2.5 μL Buffer (10X), 2.5 μL dNTPs (2 mM), 2.5 μL forward primer (10 pmol/μL), 2.5 μL reverse primer (10 pmol/μL), 0.4 μL FIREPol DNA Polymerase Solis BioDyne (5U/μL) and molecular biology quality water to complete the volume until 25 μL. For the initial PCR, SLF and S762 primers were used to amplify >2000 bp of 18S rDNA gene. For both nested-PCR, two sets of primers were used S823 and S662 primers for nested-PCR I, and S825 and SLIR primers for nested-PCR II. An additional set of optimized primers were designed and used (SKF1F and SKF1R, SKF2F and SKF2R; see Table 2). The cycling conditions for all PCR were, denaturation at 94 °C for 5 min, followed by 5 cycles of amplification at 94 °C for 30 s, 50 °C for 30 s, 72 °C for 2 min 20 s, followed by 35 cycles of amplification at 94 °C for 30 s, 52 °C for 30 s, 72 °C for 2 min 20 s and a final extension at 72 °C for 10 min. Five μL of both nested-PCR products were visualized in a 1 % agarose gel. Both nested-PCR products were sequenced in both directions with S823/S662 and S825/SLIR primers by Sanger sequencing (GENEWIZ). Sequences obtained from specimens CD8 of infected *Raja asterias* and CD44 of infected *Raja miraletus* were deposited in the GenBank database (Accession numbers: MG878996 and MG878995 respectively).

**Table 2:** List of primers used in the present study.

Name	Primer's sequence	Position	Reference
SLF F	5’_CATATGCTTGTTTCAAGGAC_3′		Maslov et al. (1996)
S762 R	5’_GACTTTTGCTTCCTCTA(A/T)TG_3′		Maslov et al. (1996)
S823 F	5'_CGAACAACTGCCCTATCAGC_3′	325–344	Maslov et al. (1996)
S662 R	5'_GACTACAAYGGTCTCTAATC_3′	1219–1238	Maslov et al. (1996)
S825 F	5'_ACCGTTTCGGCTTTTGTTGG_3′	909–928	Maslov et al. (1996)
SLIR R	5'_ACATTGTAGTGCCCGTGTC_3′	1834–1852	Maslov et al. (1996)
SKF1 F	5'_GCCATGCATGCCTCAGAATC_3	30–49	Present study
SKR1 R	5'_CTCCCTCTCCGGAATCGAAC_3′	399–418	Present study
SKF2 F	5'_CAACAGCAGGTCTGTGATG_3′	1797–1815	Present study
SKR2 R	5'_CGACTTTTGCTTCCTCTAT_3′	2123–2141	Present study

### 2.4 Phylogenetic analysis

Phylogenetic tree reconstruction was performed with newly generated 18S rDNA sequences of *T. rajae* and additional 24 selected sequences of *Trypanosoma* from NCBI GenBank including *T. avium* Danilewsky 1885, used as outgroup (Table 3). Sequences were aligned and analyzed using the ClustalW program on MEGA 11 software (Tamura et al., 2021). The best-fit model of DNA sequence evolution was selected using MEGA 11. According to the Akaibe information criterion, the General Time Reversible model including invariable sites and variation among sites (GTR + G + I) was suggested as best suiting the dataset.

**Table 3:** Collection data for 18S rDNA trypanosome sequences analyzed in this study. Newly generated sequences are in bold.

	Host	Country	GenBank	Reference
Avian trypanosome				
*T. avium*	*Xanthomyza phrygia*	Australia	KT728402	Šlapeta et al. (2016)
Turtle/Platypustrypanosomes				
*T. chelodinae*	*Emydura signata*	Australia	AF297086	Jakes et al. (2001)
*T. binneyi*	*Ornithorhynchus anatinus*	Australia	KJ867148	Paparini et al. (2014)
Marine fish trypanosomes				
*T. boissoni*	*Zanobatus atlanticus*	Senegal	U39580	Maslov et al. (1996)
*T. triglae*	*Chelidonichthys lastoviza*	Senegal	U39584	Maslov et al. (1996)
*T. pleuronectidium*	*Melanogrammus aeglefinus*	Norway	DQ016618	Karlsbakk and Nylund (2006)
*T. murmanensis*	*Hippoglossus hippoglossus* fed on by leech *Johanssonia arctica*	Norway	DQ016616	Karlsbakk and Nylund (2006)
***T. rajae***	***Raja asterias* (CD8)**	**Algeria**	**MG878996**	**Present study**
***T.*****cf.*****rajae***	***Raja miraletus* (CD44)**	**Algeria**	**MG878995**	**Present study**
*T. haploblephari*	*Haploblepharus pictus*	South Africa	MZ061638	Pretorius et al. (2021)
*T. haploblephari*	*Poroderma pantherinum*	South Africa	MZ061641	Pretorius et al. (2021)
Freshwater fish trypanosomes				
*Trypanosoma* sp. isolate TS4	*Scardinius erythrophthalmus*	Ukraine	KJ601718	Grybchuk-Ieremenko et al. (2014)
*Trypanosoma* sp. isolate R6	*Abramis brama*	Poland	AJ620554	Unpublished
*Trypanosoma* sp. isolate Ts-Ab-TB	*Abramis brama*	Czech Republic	AJ620556	Unpublished
*Trypanosoma* sp. isolate UK	*Anguila anguila*	United Kingdom	AJ620551	Unpublished
*Trypanosoma* sp. isolate El-CP	*Esox lucius*	Czech Republic	L14841	Maslov et al. (1996)
*T. cobitis*	*Noemacheilus barbatulus*	England	AJ009143	Stevens et al. (1999)
*Trypanosoma* sp. isolate CLAR	*Clarias angelensis*	Import Africa	AJ620555	
*T. carpio*	*Cyprinus carpio*	China	EF375882	Gu et al. (2007b)
*T. ophiocephali*	*Channa argus*	China	EU185634	Gu et al. (2010)
*T siniperca*	*Siniperca chuatsi*	China	DQ494415	Gu et al. (2007)
*T. granulosum*	*Anguila anguila*	Portugal	AJ620552	Unpublished
*Trypanosoma* sp. isolate TS2	*Carassius carassius*	Ukraine	KJ601715	Grybchuk-Ieremenko et al. (2014)
*Trypanosoma* sp. isolate Marv	*Cyprinus carpio*	N.a.	AJ620549	Unpublished
*Trypanosoma* sp. isolate *fulvidraco*	*Pseudobagras fulvidraco*	China	EF375883	Gu et al. (2007)
*Trypanosoma* sp.	*Micropterus salmoides*	China	MH635421	(Jiang et al., 2019)

Phylogenetic relationships were inferred using PHYML on ATGC Montpellier Bioinformatics Platform and nodal robustness evaluated by non-parametric bootstrapping (1000 replicates) (Guindon et al., 2010) and Bayesian analyses with MrBayes version 3.2.1 (Ronquist et al., 2012) for a total 3 million generations with sampling frequency of every 100 generations and the first 25 % of the trees were discarded as burn-in material prior to the construction of the consensus tree. The obtained trees were visualized in FigTree v1.4.4 and then displayed and annotated using iTOL v6.6 (Interactive tree of life) (Letunic and Bork, 2021).

### 2.5 Statistical analyses

Prevalences were calculated (Letunic and Bork, 2021). The morphometric measurements and prevalence obtained for each fish species, sex and sampling site were compared using parametric test (Student's “*t”* Test). Non-parametric test (Mann-Whitney *U* test) were used when the normality of the distributions and/or homoscedasticity (conditions for applying parametric tests) were not met, which were verified with the application of the Shapiro-Wilk test, the Levene test and the Fischer's F test of comparison of variances.

To investigate possible influences of host age on infection prevalence, we divided the rays into 4 different groups by weight: (1) up to 500 g, (2) 501–1000 g, (3) 1001–1500 g, and (4) >1500 g, and determined the prevalence in each group; prevalence was compared between groups by Fisher's Exact Test. All statistical analyses were performed with the aid of the software STATISTICA version 6.1.

## 3 Results

### 3.1 Infection details

This study was carried out with a sample of 123 skates belonging to *R. asterias* (n = 73) and *R. miraletus* (n = 50). We recorded trypanosome infection with the overall prevalence of 32.52 %, distributed according to the host species examined as 38.3 % for *R. asterias* and 24 % for *R. miraletus* (non-significant difference, P = 0.0988). Prevalence comparison between sexes (females 34.56 % vs males 28.57 %) also reveals a non-significant difference (P = 0.5025). However, there is a positive relationship between host weight and parasite prevalence; juvenile fishes have little or no parasites, the more the fishes gain weight (age), the more chances for them to become parasitized (P = 0.01, Fisher's Exact test, between host weight and parasite prevalence).

### 3.2 Morphology

Kinetoplastea Honigberg 1963.

Trypanosomatida Kent 1880.

Trypanosomatidae Doflein 1901.

*Trypanosoma* Gruby 1843.

#### *3.2.1* *Trypanosoma rajae*Laveran and Mesnil, 1902

*Type-host*: *Raja asterias* (syn. *R. punctata*), Mediterranean starry ray; *R. undulata* (syn. *R. mosaica*), the undulate ray (Rajiformes: Rajidae) (Laveran and Mesnil, 1902).

*Other hosts*: *Vertebrate hosts* (all Rajiformes, Rajidae): *Dipturus batis* Linnaeus (syn. *R. macrorynchus* Rafinesque), the common skate (Laveran and Mesnil, 1904); *Raja clavata* Linnaeus, the thornback ray (Laveran and Mesnil, 1904; Preston, 1969); *Amblyraja radiata* (Donovan) (syn. *R. radiata* Donovan), the thorny skate (So, 1972); *Leucoraja ocellata* (Mitchill) (syn. *R. ocellata* Mitchill), the winter skate (Kudo, 1923); *Leucoraja erinaceus* (Mitchill) (syn. *R. erinacea* Mitchill), the little skate (Bullock, 1958)^1^.

*Invertebrate hosts*: *Pontobdella muricata* (Linnaeus) (Robertson, 1927; Preston, 1969).

*Type-locality*: Roscoff, France, Northeast Atlantic (Laveran and Mesnil, 1902).

*Other localities*: Northeast Atlantic: Millport, Plymouth, United Kingdom (Preston, 1969). Northwest Atlantic: Newfoundland, Canada (So, 1972); Massachusetts USA (Kudo, 1923); New England, USA (Bullock, 1958). Western Mediterranean, Algeria (Present study).

*Site in host:* heart blood.

*Voucher material:* four MGG-stained blood smears deposited in the Protists collection of the Muséum National d'Histoire Naturelle, Paris, France, under the inventory numbers MNHN-IR-2020-01/02 for the infected *R. asterias* specimen CD8; including one 18S rRNA(nu18S) partial sequence. Additional material, four MGG-stained blood smears deposited at the Swedish museum of Natural history (SMNH), Stockholm, Sweden under accession number SMNH 225894–225897.

*Representative DNA sequences:* The sequence data associated with *Trypanosoma rajae* ex *R. asterias* MNHN-IR-2020-01/02 have been submitted to GenBank: nuclear 18S rRNA(nu18S) partial sequence MG878996.

Measurements indicated in Table 4. In fresh preparation, trypanosomes with an elongated body often curves and curls around itself. Movement is lively, trypanosome often changing shape quickly, sometimes bending into an S shape and sometimes curling into an O shape with both ends touching or crossing. A distinguishable undulating membrane that folds rapidly from the anterior to the posterior end present. Parasite always on the move, flagellum in front animated by movements much faster than those of body. Neither nuclei nor granulations of any kind perceptible in fresh preparations.

**Table 4:** Morphometrical data of morphotype1 of *Trypanosoma rajae*Laveran and Mesnil, 1902 from *Raja asterias* and *Raja miraletus* from Algeria, Western Mediterranean. a: Difference not significant, b: Significant difference.

	*R. asterias* (n = 21)	*R. miraletus* (n = 8)	Test	P	Difference
PK	6.18 (2.17–12.42) n = 233	6.97 (4.19–10.59) n = 106	MW	0.00	b
KN	25.32 (13.92–39.68) n = 233	24.99 (12.95–36.11) n = 100	MW	0.92	a
NA	23.55 (12.74–34.34) n = 234	23.64 (10.73–35.54) n = 100	“*t*”	0.57	a
BL	54.98 (38.5–70.47) n = 233	55.27 (35.89–68.05) n = 98	MW	0.27	a
BWN	4.43 (2.66–9.76) n = 234	5.06 (1.91–11.47) n = 104	MW	0.00	b
NuL	3.84 (1.83–6.62) n = 234	3.5 (2.05–6.65) n = 110	MW	0.00	b
NuW	2.65 (1.52–4.22) n = 234	2.6 (1.35–4.64) n = 110	MW	0.07	a
NuA	8.07(2.18–19.21) n = 234	7.25 (1.75–18.69) n = 109	MW	0.00	b
FF	21.64 (5.06–59.46) n = 234	25.01 (9.97–70.3) n = 110	MW	0.00	b
KI	1.25 (1.09–1.88) n = 233	1.28 (0.38–1.79) n = 98	MW	0.00	b
NI	1.39 (0.66–2.49) n = 233	1.38 (0.38–1.79) n = 98	MW	0.90	a
Sle	13.07 (4.22–20.,94) n = 233	12.51 (0.38–1.79) n = 98	MW	0.03	b
PN	31.5 (20.52–46.5) n = 233	31.55 (0.38–1.79) n = 98	MW	0.52	a
FI	0.40 (0.08–1.54) n = 233	0.44 (0.38–1.79) n = 98	MW	0.04	b

**Abbreviations:** PK: posterior end to kinetoplast, KN: kinetoplast to mid-nucleus, NA: mid-nucleus to anterior end, BL: body length, BWN: parasite maximum body width at nucleus, NuL: nucleus length, NuW: nucleus width, NuA: Nucleus area, FF: free flagellum length, KI: kinetoplast index = PN/KN, NI: nuclear index = PN/NA, Sle: slenderness = BL/BW, PN: posterior end to mid-nucleus, FI: flagellar index = FF/BL.

Thin blood smears stained with MGG allow an easier and more detailed description of the parasites. Trypomastigote forms with an elongated body, serpentiform to bent, C-shaped or S-shaped with 3–7 maximum flexions, most inflections at anterior end more than at posterior end.

Vacuolated cytoplasm uniformly colored, with fine chromatic granulations giving the parasite's body a purple color. Sometimes, few much larger granulations also visible. Long flagellum tightly coiled and sometimes knotted, difficult to observe in its free part. Translucent to transparent undulating membrane somewhat visible, irregularly pleated all along body. Rounded or oval, purple-stained nucleus formed by clusters of chromatin; often placed equidistant from both sides of cell body, located at union of anterior and middle thirds of body; surrounded by transparent ring; transparent ring sometimes difficult to distinguish because of granulations gathered at its periphery. Kinetoplast small, rounded in dividing forms or rod-like to nearly rectangular in non-dividing cells, located near posterior end; compact, strongly colored by MGG. Posterior end of cell is variable in shape, tapered to conical. Posterior and anterior regions less colored by MGG.

**Fig. 1:**
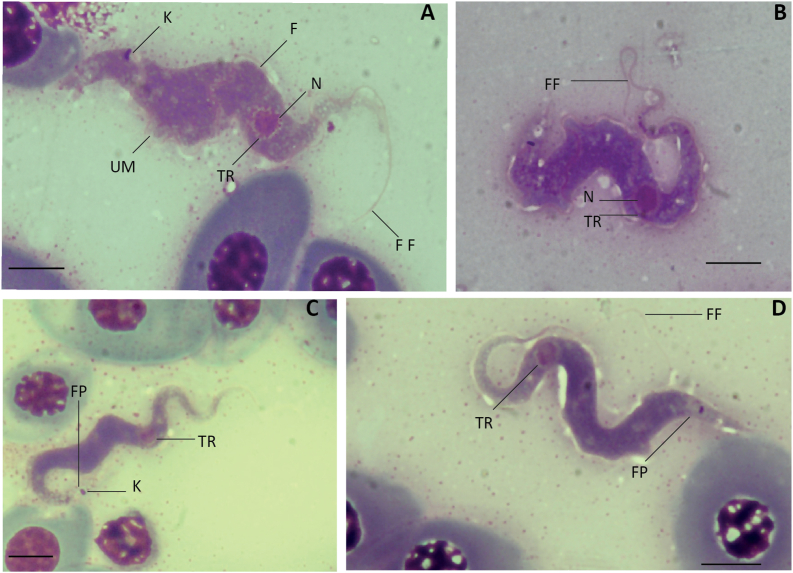
Photographs of the “elongated” morphotype (morphotype 1) of *Trypanosoma* from Rajidae from the Western Mediterranean. A, C, *Trypanosoma* cf. *rajae* ex *Raja miraletus*; B, D, *Trypanosoma rajae*Laveran and Mesnil, 1902 ex *Raja asterias*. Images from blood smears fixed with methanol and stained with MGG. Abbreviations: FF: Free Flagellum, UM: Undulating Membrane, TR: Transparent Ring, N: Nucleus, FP: Flagellar Pocket, K: Kinetoplast and F: Flagellum. Scale bar: 5 μm.

**Fig. 2:**
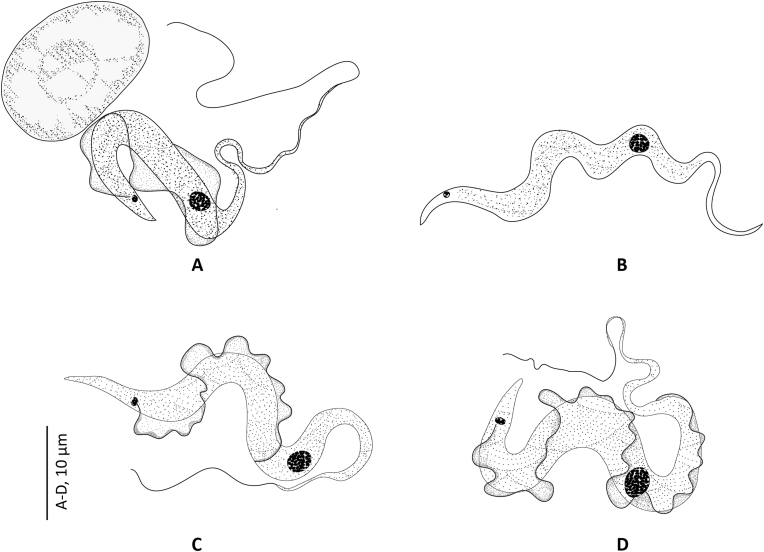
Line drawings of the “elongated” morphotype (morphotype 1) of *Trypanosoma* from Rajidae from the Western Mediterranean. Drawing from thin blood smears stained with MGG. A, B, *Trypanosoma rajae*Laveran and Mesnil, 1902 ex *Raja miraletus*. C, D, *Trypanosoma rajae*Laveran and Mesnil, 1902 ex *Raja asterias*.

**Fig. 3:**
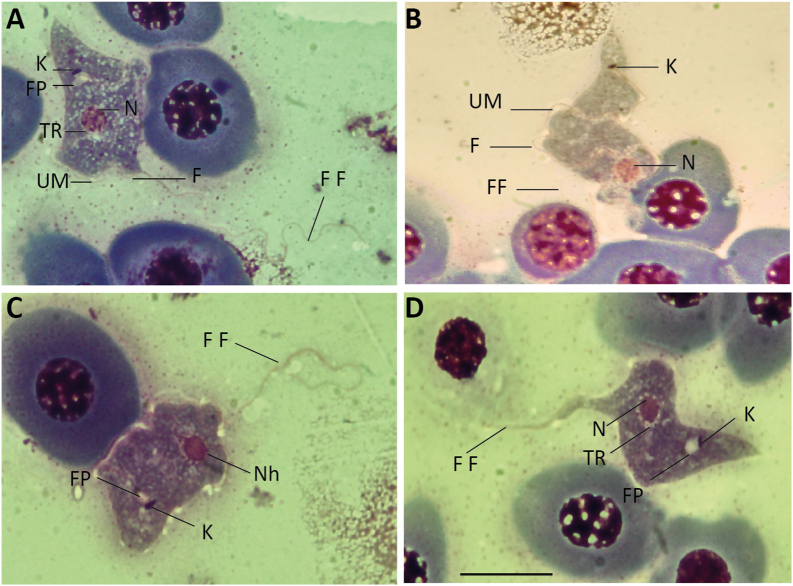
Photographs of the “stumpy” morphotype (morphotype 2) of *Trypanosoma* from Rajidae from the Western Mediterranean. A, B, *Trypanosoma rajae*Laveran and Mesnil, 1902 ex *Raja asterias*; C, D, *Trypanosoma* cf. *rajae* ex *Raja miraletus*. Images from blood smears fixed and stained with MGG. Abbreviations: K: Kinetoplast, F: Flagellum, N: Nucleus, FF: Free Flagellum, FP: Flagellar Pocket and TR: Transparent Ring. Scale bar: 10 μm.

**Fig. 4:**
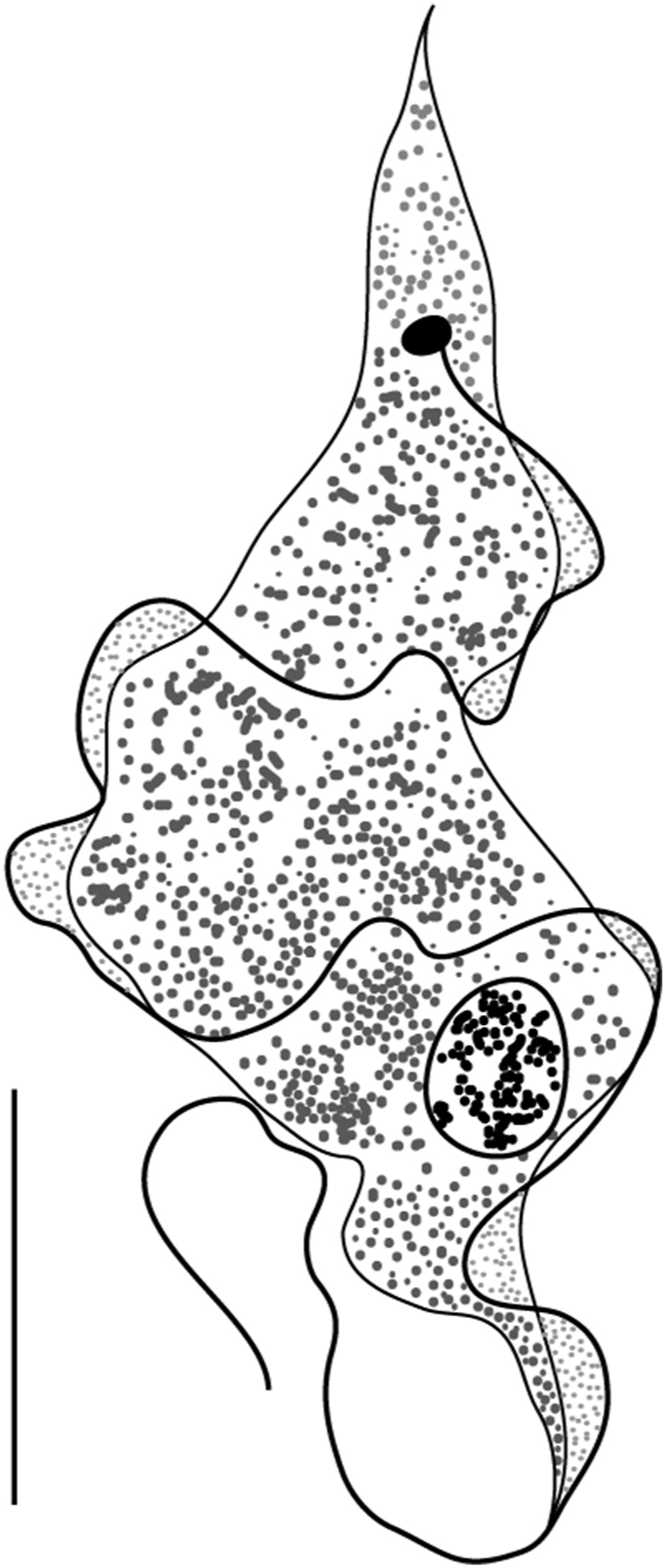
Line drawings of the “stumpy” morphotype (morphotype 2) of *Trypanosoma* ex *Raja asterias* from the Western Mediterranean. Drawing from thin blood smears stained with MGG.

In all the smears examined, two morphotypes were observed: morphotype 1, the “elongated” form, and morphotype 2, the “stumpy” form. Morphotype 1: elongated, thin and fusiform (Fig. 1, Fig. 2), mostly S-shaped. Morphotype 2: wider (Fig. 3), and with a distinctive stumpy appearance (Fig. 4). Morphotype 2 shows the cytoplasm to be more granular and/or vacuolated, and nucleus with a granular appearance associated to with increased condensation of chromatin. No striations in the cytoplasm or the nuclear zone were visible in either morphotypes. Measurements of the different morphological parameters of both morphotypes of *T. rajae* specimens, between both hosts, showed a significant difference by the Mann-Whitney non-parametric statistical test, except for the nucleus area NuA (Table 4).

**Fig. 5:**
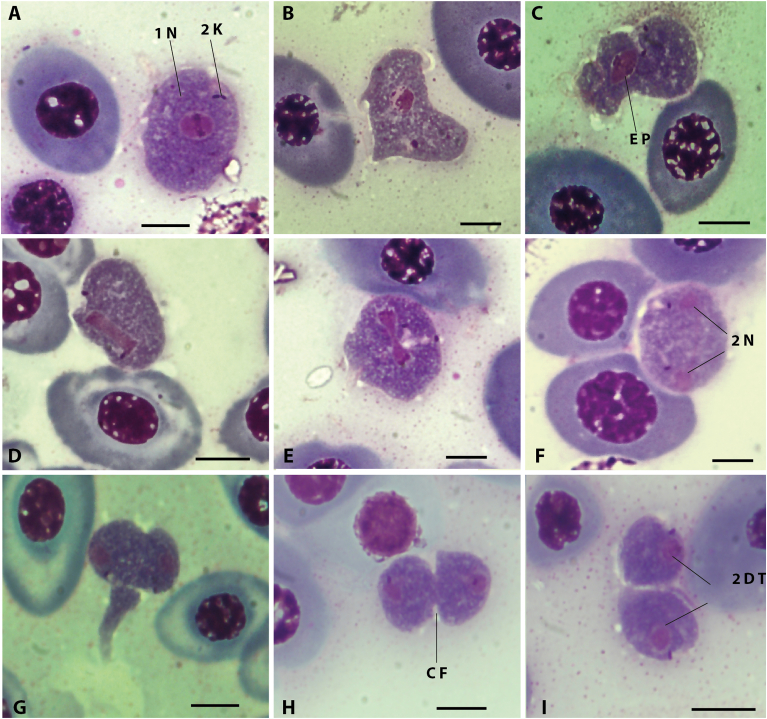
Stages of trypanosome cell division on thin smears of *Trypanosoma rajae*Laveran and Mesnil, 1902 examined from the blood of *Raja asterias*, stained with MGG. A, 1K1N stage with duplicating kinetoplast. B, 2K1N stage. C-E, 2K1N stages with dividing nucleus. F, 2K2N stage. G-I, Cytokinesis of 2K2N stages. Abbreviations: N, Nucleus. K, Kinetoplast. EP, Equatorial plate. CF, Cleavage furrow. DT, Daughter trypanosomes. Scale bar: 5 μm.

Dividing parasites were commonly observed in parasitized fish, in *R. asterias* 12/28 (42.85 %) and in *R. miraletus* 5/12 (41.66 %), giving a total of 17/40 or 42.5 %; on stained blood smears (e.g., 29.1 % of trypanosomes observed on smears of *R. miraletus* specimen CD44 were entered in division, n = 127). They generally exhibited a rounded shape (Fig. 5 A) with some of them showing a more or less long and thin extremity (Fig. 5 B). Dilated stumpy dividing forms looking like intermediate forms between stumpy form and round division forms were also observed (Fig. 5B and C). Cell division begins by the duplication of the kinetoplast followed by the nucleus division (Fig. 5A–F). The rod-like kinetoplast widens slightly and divides into two small, rounded parts of equal size closely associated to two flagellar pockets (stage 2K1N). In the nucleus, chromatin condensates (Fig. 5A and B), especially at two poles of the nucleus that will guide the nucleus division plan (equatorial plate) leading to an elongation of the nucleus (Fig. 5C and D) and setting up of a division furrow at equal parts of both poles (Fig. 5 E) generating to two individualized nuclei (Fig. 5 F) (stage 2K2N). After the formation of the two daughter nuclei, cytokinesis occurs (Fig. 5G–I) with the division of the cell body by a cleavage furrow, and ends with the formation of two daughter trypanosomes, the size and shape of which are identical (Fig. 5 I). Cell shape did not allow us to make conclusive observations on the presence and length of the flagella during division.

#### *3.2.2* *Trypanosoma* cf. *rajae*

*Host*: *Raja miraletus* (Rajiformes: Rajidae) (present study).

*Locality*: Bouharoune and Cap Djinet, Algeria, Western Mediterranean (present study).

*Site in host*: heart blood.

*Voucher material:* One MGG-stained blood smear deposited in the Protists collection of the Muséum National d'Histoire Naturelle, Paris, France, under the inventory numbers MNHN-IR-2020-01/02 for the infected *R. miraletus* specimen CD44; with corresponding 18S rRNA(nu18S) partial sequence.

*Representative DNA sequences:* The sequence data associated with *Trypanosoma* cf. *rajae* ex *R. miraletus* MNHN-IR-2020-01/02 have been submitted to GenBank: nuclear 18S rRNA(nu18S) partial sequence MG878995.

General morphology similar to that of *T. rajae* described above in *R. asterias*, with an elongated and fusiform body measuring 55.27 μm (35.89–68.05), slightly wider with 5.06 μm (1.91–11.47). Cytoplasm uniformly colored, containing larger granulations sometimes visible. Round or oval core with area of 7.25 μm (1.75–18.69), located in anterior third of body with distance from mid-nucleus to anterior end of 23.64 μm (10.73–35.54).

Undulating membrane running all along body with flagellum lining ending with rather large free part measuring 25.01 μm (9.97–70.3). Kinetoplast small, strongly colored, rod-shaped, located in posterior third near tip of 6.97 μm (4.19–10.59). Distance of kinetoplast determining its conical shape.

#### 3.2.3 Remark

In Fig. 7, we mapped the distribution of these 3 host fishes (Fischer et al., 1987; FAO, 1994; Horton et al., 2020; Froese and Pauly, 2021). *Raja undulata* is known from the Eastern Atlantic: from the south of Ireland and England to Senegal, including the Western Mediterranean and the Canary Islands and also from the Eastern Mediterranean. *Raja asterias* is endemic to the Mediterranean but can spread to the Strait of Gibraltar, northern Morocco, and possibly southern Mauritania, it was also recorded in the Eastern Atlantic. *Raja miraletus* is known from the Eastern Atlantic: northern Portugal and throughout the Mediterranean as far as Madeira and South Africa, also in the southwestern Indian Ocean (Su et al., 2014). The distributions of the host species overlap mainly in the Eastern Atlantic and throughout the Mediterranean.

### 3.3 Phylogenetic analysis

**Fig. 6:**
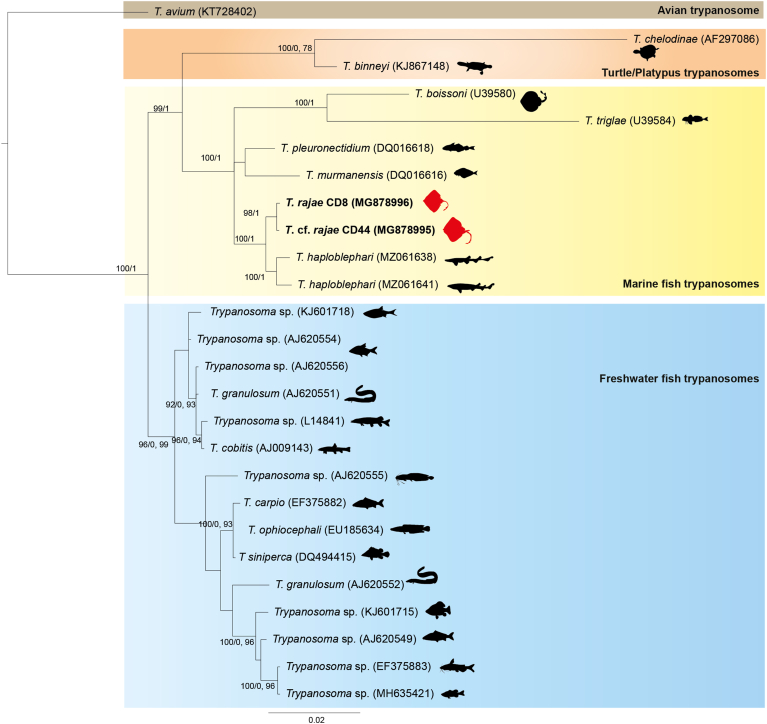
Molecular phylogenetic analysis by Maximum-Likelihood methods with GTR + G + I model, maximum likelihood bootstrap values (1000 replicates) and Bayesian posterior probabilities are indicated on branches. Partial 18S rRNA gene sequences of *Trypanosoma* parasites of fishes or aquatic vertebrates were retrieved from GenBank for phylogenetic reconstruction. The phylogenetic tree was rooted using avian trypanosomes. Node values less than 50 % were not displayed. Newly generated sequences are framed in red.

Partial 18S rRNA gene sequences of trypanosomes were successfully amplified from blood smears of one infected *R. asterias* specimen (CD8) and one infected *R. miraletus* specimen (CD44) and sequenced. 2071 and 2112 bp sequences were obtained and deposited under the GenBank accession numbers MG878996 and MG878995, respectively. Both sequences showed ∼99.76 % pairwise identity (Fig. 6). Blast analyses revealed that the most similar sequences have been previously detected in fish or leech trypanosomes, with the best score (98 % identity) for a sequence of *T. murmanense* Nikitin, 1927 (DQ016616), a trypanosome isolated from the fish leech *Johanssonia arctica* (Karlsbakk et al., 2005; Karlsbakk and Nylund, 2006).

**Fig. 7:**
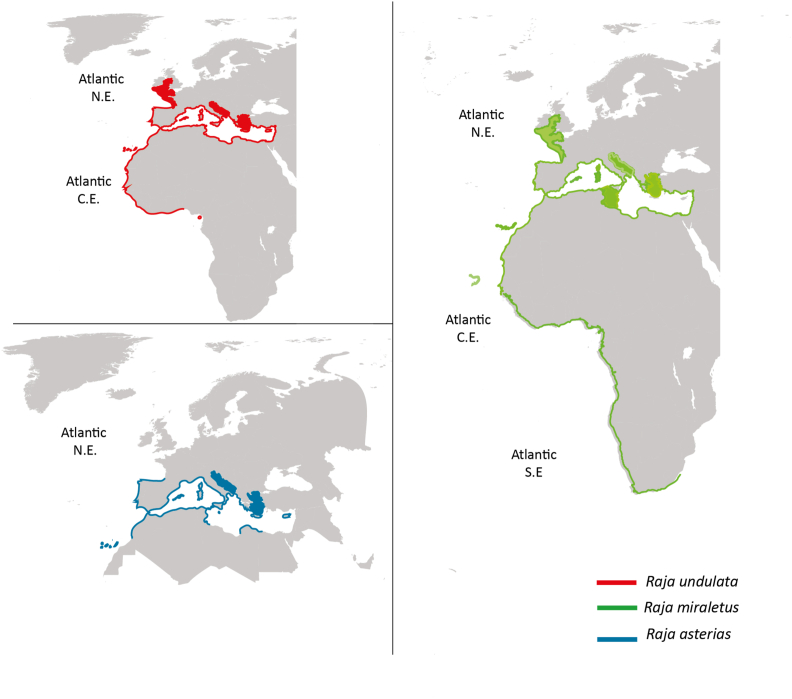
Biogeographic distribution of *Raja undulata* and *Raja asterias*, type-hosts of *Trypanosoma rajae*Laveran and Mesnil, 1902; and of *Raja miraletus* reported herein as host for *Trypanosoma* cf. *rajae*.

Phylogenetic analysis was performed using partial 18S rDNA sequences of trypanosomes of freshwater and marine fishes, fish leeches, turtle, platypus and an avian trypanosome as outgroup (Fig. 6). Trypanosomes from *R. asterias* and *R. miraletus* (CD8 and CD44) grouped together and appear to be sister to *T. haploblephari*. The marine fish trypanosomes clade appears distinctive from the freshwater/turtle/platypus trypanosome group and with the freshwater fish trypanosomes as a sister group. Amongst marine trypanosomes, the closest species were *T. murmanense* (isolated from *Hippoglossus hippoglossus* fed on by the leech *Johanssonia arctica*) and *T. pleuronectidium* Robertson, 1906 isolated from the haddock *Malanogrammus aeglefinus* (Linnaeus) (Karlsbakk and Nylund, 2006).

## 4 Discussion

### 4.1 Host-specificity and morphological variability of *Trypanosoma rajae*Laveran and Mesnil, 1902

Although more species of trypanosomes have been formally described from marine teleost fishes than from elasmobranchs, research on trypanosomes in elasmobranchs remains limited and underexplored. To date, 32 species have been described from teleosts, compared to only 12 from elasmobranchs. Of these, eight teleost-derived species have been molecularly characterized (Karlsbakk and Nylund, 2006; Su et al., 2014; Le Roux et al., 2025), whereas only two elasmobranch-derived species have undergone molecular characterisation (Maslov et al., 1996; Pretorius et al., 2021). The parasitic kinetoplastids detected in the blood of *R. asterias* and *R. miraletus* collected off the Algerian coast in the Western Mediterranean belong to the genus *Trypanosoma* Gruby, 1843. Several trypanosome species have been described in elasmobranch fishes, including representatives of the orders Carcharhiniformes (Laveran and Mesnil, 1904; Morillas et al., 1987; Yeld and Smit, 2006), Lamniformes (Laveran, 1908), Myliobatiformes (Ranque, 1973 *in* Yeld and Smit, 2006; Burreson, 1989b), Rajiformes (Laveran and Mesnil, 1902, 1904; Neumann, 1909; Laird and Porter, 1951; Bacigalupo and De la Plaza (1948) *in* Yeld and Smit, 2006), Torpediniformes (Sabrazes and Muratet (1908) *in* Yeld and Smit, 2006), and Orectolobiformes (Burreson, 1989). Many of these descriptions date back more than a century and were based primarily on morphology, highlighting the need for revision using modern approaches. This study makes an important contribution by redescribing an elasmobranch *Trypanosoma* species with greater morphological detail and providing molecular data, thus enhancing taxonomic resolution and adding to the broader trypanosome phylogeny. These results highlight the importance of revisiting historical species descriptions to better understand parasite diversity and host specificity in marine elasmobranchs.

Table 5reports the morphometric data measured on the specimens infecting *R. asterias* and *R. miraletus* in the present study and those reported from these described species, closest to the hosts studied, as well as *T. pleuronectidium* Robertson, 1906 and *T. murmanense* Nikitin, 1927 which are found to be closest phylogenetically to the *T. rajae* of the present study. Our specimens can be easily distinguished mainly from: *T. gargantua* Laird, 1951; *T. haploblephari* Yeld and Smit, 2006 and *T. humboldti* Morillas et al., 1987 by the absence of longitudinal striations (myonemes). Note that Pretorius et al. (2021) demonstrated that *T. haploblephari* clustered with *T. rajae*, exhibiting low genetic divergence despite clear morphological differentiation and distinct host associations, with the former parasitizing sharks and the latter rays. In light of the documented pleomorphism in *T. rajae*, the authors adopted a cautious taxonomic approach. The present study reinforces the complexity within the genus *Trypanosoma* and underscores the limitations of relying solely on molecular divergence for species delimitation. Our findings support the recognition of *T. haploblephari* and *T. rajae* as distinct species based on consistent morphological traits and strict host specificity. These results highlight the necessity of an integrative taxonomic framework that considers morphology, host range, and molecular data for accurate species delineation within this group (see Table 6).

**Table 5:** Comparative morphometric data of *Trypanosoma rajae*Laveran and Mesnil, 1902 from *Raja asterias* and *Raja miraletus* from Algeria, Western Mediterranean and *Trypanosoma* spp. from Elasmobranchii. All measurements are μm. 1 Considered synonym of *Trypanosoma rajae*.

Parasite	Host species	L T	B L^a^	F F	B W	NuL	NuW	P K	N K	Str	References
*T. giganteum*	*R. microcellata*	–	81 ± 9 (70–90)	10 ± 1 (10–12)	9 ± 2 (6–11)	4 ± 1 (3–5)	4 ± 1 (3–5)	19 ± 4 (4–25)	39 ± 5 (30–45)	NO	Aragort et al. (2005)
	*R. brachura*										
	*Raja* spp.										
*T. rajae*	*Leucoraja ocellata*	–	30–35	6–8	2.3	3	2.2	6	–	Yes	Kudo (1923)
*T. rajae*	*R. asterias*	75–80	–	20	6	–	–	–	–	NO	Laveran and Mesnil (1902)
	*R. undulata*										
*T. rajae*	*Raja* sp.	67	–	13 (10–15)	5.2	–	–	–	–	NO	Minchin and Woodcock (1910)
*T. rajae*	*R. erinacea*	–	42–58.9	11–16.7	–	–	>1.8 (3.8–6.3)	–	–	NO	Laird and Bullock (1969)
	*R. radiata*										
*T. variabile*^1^*(large form)*	*R. punctata*	90–100	80–85	20–25	4–7	–	–	–	–	NO	Neumann (1909)
*T. variabile*^1^*(small form)*	*R. punctata*	40	30–33	8	1.5–5	–	–	–	–	NO	Neumann (1909)
*T. scyllii*	*Scyliorhynus canicula*	70–75	–	14	5–6	–	–	–	–	NO	Laveran and Mesnil (1902)
	*S. stellaris*										
*T. scyllii (large form)*	*S. cunicula*	70.6	58.61	12.02	6.35	5.05	5.05	9.97	–	Yes ∗	Pulsford (1984)
*T. scyllii (small form)*	*S. cuniculu*	67.6	54.07	13.5	3.69	3.69	2.46	9.22	–	Yes∗	Pulsford (1984)
*T. gargantua (large form)*	*Dipturus nasutus* (syn*. Raja nasuta*)	114.7	–	–	10.1–14.3	7.8	6	16.5 % BL	–	Yes ∗∗	Laird and Porter (1951)
*T. gargantua (small form)*	*D. nasutus* (syn. *Raja nasuta*)	66.7–131.1	–	–	4.6–13.7	3.9–9.3	3.3–6.7	13–25 % BL	–	Yes ∗∗	Laird and Porter (1951)
*T. haploblephari*	*Haploblepharus pictus*	–	70 (53.7–99.4)	N.A.	17.4 (12.6–24.3)	6.5 (5.2–8.8)	6.5 (4.7–8.8)	–	–	Yes∗∗∗	Yeld and Smit (2006)
*T. taeniurae*	*Taeniura lymma*	–	55	>9	4	3.5	4.5	–	–	Yes	Burreson (1989)
*T. marplatensis*	*Sympterygia bonapartii* (syn. *Psammobatis microps*)	–	60–65	>15	10–12	7.2	4.2	–	–	NO	see Yeld and Smit (2006)
*T. carchariasi*	*Carcharias* sp.	60–70	–	–	–	–	–	–	–	–	Laveran (1908)
*T. mackerrasi*	*Hemiscyllium ocellatum*	–	125	–	15	7	10	–	–	Yes	Burreson (1989)
*T. boissoni*	*Zanobatus schoeleini*	–	60.1 (45–67)	1.3 (0–7)	4.6 (3.3–7)	N.A.	N.A.	–	–	NO	see Yeld and Smit (2006)
*T. humboldti*	*Schroederichthys chilensis*	87 (78–93)	–	6.8 (5–11)	7.4 (4–10)	5.3 (5–6)	5.3 (5–6)	18.8 (16–25)	36.6 (31–46)	Yes	Morillas et al. (1987)
*T. rajae*	*R. asterias*	–	54.98 (38.5–70.47)	21.64 (5.06–59.46)	4.43 (2.66–9.76)	3.84 (1.83–6.62)	2.65 (1.52–4.22)	6.18 (2.17–12.42)	25.32 (13.92–39.68)	NO	present study
*T.* cf. *rajae*	*R. miraletus*	–	55.27 (35.89–68.05)	25.01 (9.97–70.3)	5.06 (1.91–11.47)	3.5 (2.05–6.65)	2.6 (1.35–4.64)	6.97 (4.19–10.59)	24.99 (12.95–36.11)	NO	present study

**Abbreviations:** NO: Not Observed, –: Not Available, ∗: Nucleus zone, ∗∗: All along the body, ∗∗∗: Longitudinal striations sometimes visible on the more deeply stained, larger specimens, particularly over the nuclear area. ^a^ Body length does not include the free flagellum.

**Table 6:** Comparative data of *Trypanosoma rajae*Laveran and Mesnil, 1902 from *Raja asterias* from Algeria, Western Mediterranean with the previous records of *Trypanosoma rajae* in the literature.

Parameters	*T. rajae*	*T. rajae*	“T” test	*T. rajae*	‘’T″ test
Source	Present study	Kudo (1923)		Laveran and Mesnil (1902)	
BL	54.98	30–35	No overlapping	55–60	Overlapping
FF	21.64	6	P = 0.0000^a^	20	P = 0.0000^a^
BW	4.43	–	–	6	P = 0.0000^a^
NuL	3.84	3	P = 0.0000^a^	–	–
NuW	2.63	2.2	P = 0.0000^a^	–	–
P K	6.18	6	P = 0.029^a^	–	–
NK	25.32	–	–	–	–

a Significant difference.

Our specimens can further be differentiated from *T. giganteum* Neumann, 1909; *T. gargantua* Laird and Porter, 1951 and *T. mackerrasi* Yeld and Smit, 2006 by the length of the body being larger; the length of the free flagellum (which is absent in *T. mackerrasi* and in *T. haploblephari*); the width of the body mainly compared to *T. giganteum*; and the position of the nucleus which is situated in the middle of the body in *T. giganteum* and *T. humboldti* Morillas et al., 1987, whereas it can be observed in the union of the anterior and middle third of the body in our specimens.

The overall dimensions of our *Trypanosoma* specimens are close to those described by Laveran and Mesnil (1902) for *T. rajae*, body length (55–60 μm vs. 54.98 μm) and width (6 μm vs. 4.43 μm), flagellar length (20 μm vs. 21.64 μm) and PK distance (4–10 μm vs. 2.17–12.42 μm). However, the localities are distinct (Atlantic Ocean vs. Mediterranean Sea). They differ however from *T. rajae* described by Kudo (1923) by the host (*R. ocellata* vs. *R. asterias*) and the locality (Atlantic Ocean vs. Mediterranean Sea). Moreover, Kudo's trypanosomes are smaller (30–35 μm vs. 38.5–70.47 μm) with a shorter flagellum (6–8 μm vs. 5.06–59.46 μm) and the presence of striations all along the body which are absent in the present specimens. They also differ from *T. rajae* reported by Minchin and Woodcock (1910) in *Raja* sp. in body width of the order of 5.2 μm vs. 4.43 μm and free flagellum as indicated in Table 4; Laird and Bullock (1969) in body length (42–58 μm) vs. 54.98 μm (38.5–70.47 μm), with a shorter flagellum (11–16.7 μm) vs 21.64 μm (5.06–59.46 μm). Also, hosts (*R. erinacea* and *R. radiata* vs. *R. asterias*) and localities (Atlantic Ocean vs. Mediterranean Sea) are different.

Our specimens reveal certain points of similarity and dissimilarity with *T. scyllii* infecting *Scyliorhinus canicula* and *S. stellare* (Pulsford, 1984) by the length of the body which is 54–61 μm vs. 54.98 μm (38.5–70.47 μm), the width of the body 5–6 μm vs. 2.66–9.76 μm and a shorter flagellum 14 μm vs. 21.64 μm. It is important to note that in the original description of *T. scyllii* by Pulsford (1984), the FF, Nul, and PK differs from the specimens examined in the present study (Table 5). Based on morphometric characters, *T. rajae* and *T. scyllii* are thus the most closely morphologically comparable species to our specimens. In their original descriptions, *T. rajae* and *T. scyllii* are essentially distinguished by two features: kinetoplast rod-like in *T. rajae* while it is more oval to rounded in *T. scyllii*; the undulating membrane is broad and very wrinkled in *T. scyllii* (Laveran and Mesnil, 1904). In our specimens, the posterior end showed variability in shape from conical to tapered, corresponding more to that which was described for *T. rajae* than for *T. scyllii*. In addition, *T. scyllii* was described from a different elasmobranch host (*Scyliorhinus*, a shark vs. *Raja*, a skate).

Host specificity is a particularly relevant issue for interpreting the identity of *T. rajae*. As previously reported by Preston (1969) and So (1972) this species is known for its high polymorphism, which complicates its identification based solely on morphological characters. In the present study, we observed notable differences between trypanosomes found in *R. asterias* and *R. miraletus*. While these differences could suggest the presence of distinct species, it is also plausible that they reflect the high degree of intraspecific polymorphism previously described in *T. rajae*. Similar patterns of variability have been documented in other fish trypanosomes (Lom and Dyková, 1992). These observations highlight the challenges inherent in relying solely on morphology and reinforce the value of combining morphological and molecular approaches to clarify species boundaries and host associations. Hence, as comparison of our specimens with the original description of *T. rajae* gave no conclusive difference, we consider our specimens infecting *R. asterias* and those originally described by Laveran and Mesnil (1902) as *T. rajae* to be conspecific.

As shown in Fig. 7*, R. undulata* occurs in the Eastern Atlantic and the Western and Eastern Mediterranean and the Canary Islands; *R. asterias* is mostly Mediterranean and has been found in the Eastern Atlantic; while *R. miraletus* is distributed from northern Portugal through the Mediterranean, down to Madeira and South Africa, and also occurs in the southwestern Indian Ocean. These species overlap mainly in the Eastern Atlantic and Mediterranean, potentially allowing *T. rajae* to spread among them via leeches. While leeches are generally not host-specific, marine fish trypanosomes are considered host-specific to the fish rather than the leech vector. As such, we conservatively refer to the trypanosomes found in *R. miraletus* as *T*. cf. *rajae* pending further confirmation.

### 4.2 Divisional stages of trypanosomes

The two morphotypes (“elongated” and “stumpy”) distinguishable in our specimens highlight morphological differences and illustrate different physiological states of the same *Trypanosoma* species.

The regular occurrence of division forms in our samples is unusual for a marine fish trypanosome. Division of bloodstream forms seems a rare event (Burreson, 2007). Laveran and Mesnil reported the absence of dividing forms in their observations of *T. rajae* (Laveran and Mesnil, 1902, 1904, 1912). It is important to note that the observed patterns may be influenced by factors such as limited host sample size or seasonal variation in parasite prevalence. Future studies incorporating broader temporal sampling and increased host representation would be valuable for confirming the consistency of these findings.

Rare dividing blood forms of *T. variabile* (Gu et al., 2007) were reported in *R. asterias* (syn. *R. punctata*) by Neumann (1909). Dividing forms of species identified as *T. rajae* were commonly found in the common marine leech *Pontobdella muricata* (Linnaeus) (Robertson, 1907, 1909, 1927) or in cultures of trypanosomes from the infected blood of *R. clavata* (Preston, 1969). In the current study, dividing parasites were frequently observed on stained blood smears (up to 29 % of parasites) collected from *R. asterias* including CD8 and offer the first attempt to describe the division pattern of *T. rajae* in their vertebrate type-host. No dividing slender trypomastigote forms and typical patterns of division as reported for *T. pacifica* (Burreson and Karlsbakk, 2007) or *T. brucei* (Zhou et al., 2014) were observed.

Dividing forms were observed only in rounded cells and followed the typical pattern of division: kinetoplast duplication, followed by nuclear division and cytokinesis to give rise to two daughter trypanosomes. Unfortunately, cell shape did not allow us to make conclusive observations on the presence and length of the flagella, and the emergence of the new flagellum during division. Some diving forms showed a more or less long and thin extremity, and some enlarged stumpy dividing forms looking like intermediate forms between thin trypomastigotes and round division forms were also observed. These observations suggest that round forms originate from a process of cellular differentiation induced by still unknown factors. In addition, how round daughter cells would give rise to slender trypomastigotes needs to be clarified. Nor can it be excluded that these division forms would be persistent forms of a recent infection by an infected leech, since dividing forms of species supposed to be *T. rajae* in the common marine leech *Pontobdella muricata* (Robertson, 1907, 1909, 1927), are very similar to the bloodstream dividing forms observed in our study. In particular, Robertson (1927) described dividing forms as rounded cells with duplicated kinetoplasts, centrally located nuclei, and a clear furrow indicative of cytokinesis. He also noted the presence of occasional narrow elongated extensions at one pole of the cell.

Based on the morphological similarity of this study's trypanosome to *T. rajae*, and the type-host being in common (*R. asterias*) (see Table 7), we assign the present study's trypanosome to *T. rajae*. Unfortunately, molecular data is not available from the type-host in the type-locality for *T. rajae*, but given the proximity of the type-location and distribution of the type-host, it is likely that the present study's trypanosome is *T. rajae*.

**Table 7:** Previous records of *Trypanosoma rajae*Laveran and Mesnil, 1902*.* Type-host and type-locality are indicated in bold.

Host	Locality	Source
***Raja asterias*****Delaroche. (syn.*****R. punctata*****Risso.)**	**Roscoff, France, NEA**	Laveran and Mesnil (1902)
***Raja undulata*****Lacepède (syn.*****R. mosaica*****Lacepède.)**		
*Raja asterias* (syn. *Raja punctata*)	Roscoff, France, NEA	Laveran and Mesnil (1904)
*Raja undulata* Lacepède (syn. *R. mosaica*)		
*Dipturus batis* Linnaeus. (syn. *R. macrorynchus* Rafinesque.)		
*Raja clavata* Linnaeus.		
*Raja* sp.	Rovigno, Croitia, Adriatic Sea	(Minchin and Woodcock, 1910)
*Raja clavata* Linnaeus.	Millport, Plymouth, United Kingdom, NEA	(Preston, 1969)
*Pontobdella muricata* (Linnaeus.)	Millport, Plymouth, United Kingdom, NEA	Preston (1969)
*Pontobdella muricata* Linnaeus.	Not available	Robertson (1927)
*Amblyraja radiata* (Donovan.) (syn. *R. radiata* Donovan.)	Newfoundland, Canada, NWA	So (1972)
*Leucoraja ocellata* (Mitchill.) (syn. *R. ocellata* Mitchill.)	Massachusetts USA, NWA	Kudo (1923)
*Leucoraja erinacea* (Mitchill.) (syn. *R. erinacea* Mitchill.)	New England, USA, NWA	Bullock (1958)

**Abbreviations:** NEA, Northeast Atlantic. NWA, Northwest Atlantic.

## 5 Conclusion

This study represents the first integrative taxonomic investigation of *T. rajae*, combining morphological observations and molecular data. Our findings confirm the presence of *T. rajae* in *Raja asterias* off the Algerian coast, extending the known geographic distribution of this species to the Western Mediterranean. The identification of trypanosomes from *Raja miraletus* displaying minor morphometric differences but high genetic similarity suggests that these parasites may belong to *T. rajae* or a closely related lineage, leading us to adopt a conservative classification as *T*. cf. *rajae*.

Moreover, we provide the first documented observations of *T. rajae* division stages in the host blood, contributing to a better understanding of its biology. Phylogenetic analysis positions *T. rajae* within the broader diversity of aquatic trypanosomes, reinforcing its relationship with other species infecting marine and freshwater hosts worldwide. This work highlights the importance of integrating morphology and molecular tools for elucidating the diversity and distribution of blood parasites in marine hosts. Future studies should further explore the life cycle, host specificity, and transmission pathways of *T. rajae* to refine its taxonomic placement and ecological significance.

## CRediT authorship contribution statement

**Sabrina Kefil:** Writing – original draft, Software, Methodology, Investigation, Funding acquisition, Formal analysis, Data curation, Conceptualization. **Linda Duval:** Software, Methodology, Formal analysis. **Amandine Labat:** Software, Methodology, Formal analysis. **Chahinez Bouguerche:** Writing – review & editing, Writing – original draft, Visualization, Validation, Software, Methodology, Investigation, Formal analysis, Conceptualization. **Nadia Kechemir-Issad:** Writing – review & editing, Writing – original draft, Visualization, Validation, Supervision, Software, Resources, Project administration, Methodology, Investigation, Funding acquisition, Formal analysis, Data curation, Conceptualization.

## Ethical approval

All applicable institutional, national, and international guidelines for the care and use of animals were followed.

## Funding

This work was jointly funded by: 1. La Direction Générale et de la Recherche Scientifique (DGRSDT) in farmwork of the project “Vecteurs et Maladies vectorisées en zones humides D00L02UN160120220001. 2. Unité Molécules de Communication et Adaptation des Microorganismes (MCAM, UMR7245), Muséum National d'Histoire Naturelle, Paris, France. 3. Département des Sciences de la Nature et de la Vie, Faculté des Sciences, Université BenYoucef BENKHEDDA, Alger, Algérie. 4. Université des Sciences et de la Technologie Houari Boumediene, Faculté des Sciences Biologiques, Laboratoire de Biodiversité et Environnement: Interactions - Génomes, BP 32, El Alia Bab Ezzouar, Alger, Algérie. Open acess funding provided by Swedish Museum of Natural History.

## Conflict of interest

None.

## Footnotes

1 Note that this host is in the Northwest Atlantic.
